# Beyond the Plate: Evidence-Based Psychotherapies for Eating Disorders - A Transdiagnostic Comparison

**DOI:** 10.1192/j.eurpsy.2025.1401

**Published:** 2025-08-26

**Authors:** R. M. Lopes, A. F. Silva, A. C. Rodrigues, A. C. Matias-Martins, F. M. A. Santos, P. M. F. Coelho, T. A. A. Vieira, V. S. Melo

**Affiliations:** 1 Psychiatry and Mental Health Department, Unidade Local de Saúde do Médio Tejo, Tomar, Portugal

## Abstract

**Introduction:**

Eating disorders (EDs), including binge-eating disorder (BED), bulimia nervosa (BN), and anorexia nervosa (AN), represent serious mental health conditions characterized by disturbances in eating behavior and body image concerns. These disorders significantly impair health, psychosocial functioning, and quality of life. Evidence-based psychotherapies have shown effectiveness in treating EDs.

**Objectives:**

This study aims to evaluate and compare the effectiveness of various psychotherapies for treating BED, BN, and AN, with a focus on both short-term and long-term outcomes. A secondary objective is to assess the applicability of transdiagnostic psychotherapy approaches across different EDs.

**Methods:**

A literature review was conducted using articles from PubMed, focusing on the terms “eating disorders”, “evidence-based psychotherapy”, “cognitive-behavioral therapy”, and “interpersonal psychotherapy”. The selection prioritized the most relevant clinical trials and meta-analyses.

**Results:**

Cognitive-behavioral therapy (CBT) consistently demonstrated significant short-term effects in reducing binge-eating episodes and EDs psychopathology, particularly in BED and BN. It outperformed both inactive controls (e.g., wait-lists) and other psychotherapies. Long-term, CBT continued to show sustained improvements in symptom reduction, particularly for BED, though it was less effective for BN and AN.

For the treatment of AN, most guidelines recommended psychological interventions, particularly family-based therapy (FBT) for younger patients. CBT and structured therapies like Maudsley Anorexia Nervosa Treatment for Adults (MANTRA) were also recommended. Interpersonal psychotherapy (IPT) received limited support due to insufficient evidence.

In BN, CBT was widely endorsed as the first-line treatment. IPT was recommended as an alternative, noted for its slower symptom \reduction but equivalent long-term efficacy. FBT was recommended for younger patients.

For BED, CBT was consistently recommended as the first-line treatment, with growing evidence supporting guided CBT self-help. IPT was recommended by several guidelines as an alternative.

**Conclusions:**

CBT was the most consistently recommended treatment for all EDs, particularly for BN and BED, offering faster symptom reduction, higher remission rates, and better long-term outcomes. While IPT is a viable alternative, particularly for BED, it generally takes longer to achieve comparable results. The findings emphasize the need for more personalized treatment approaches and the exploration of adjunctive therapies to improve outcomes, especially for AN. Further research is required to refine therapy selection and address the distinct challenges posed by different ED subtypes, particularly in achieving long-term treatment success.

**Disclosure of Interest:**

None Declared

